# Perceived Algorithmic Care Intensity and Older Adults’ Autonomy in Community Smart Care: Mixed Methods Study

**DOI:** 10.2196/94559

**Published:** 2026-07-17

**Authors:** Xianghui Huang

**Affiliations:** 1 Logistics and E-commerce College, Zhejiang Wanli University, 8 Qianhu S Rd, Ningbo, Zhejiang, 315104, China, 86 13732496278

**Keywords:** community smart eldercare, perceived algorithmic care intensity, decisional substitution, perceived surveillance, perceived autonomy

## Abstract

**Background:**

As smart older adult care shifts from basic information support to more continuous algorithm-driven care, concerns have grown about whether such systems support older adults’ independence or weaken their sense of autonomy. Although previous research has focused mainly on technology acceptance, less is known about how algorithmic care shapes perceived autonomy and through which psychological pathways this occurs.

**Objective:**

This study examined whether perceived algorithmic care intensity reduces older adults’ perceived autonomy in community smart care settings, whether decisional substitution and perceived surveillance mediate this relationship, and whether digital literacy moderates these effects.

**Methods:**

A 3-stage design was adopted. First, a qualitative prestudy with 15 older adults was conducted to refine the focal constructs and measures. Second, a vignette-based experiment with 233 valid participants tested the effects of algorithmic care intensity on decisional substitution, perceived surveillance, and perceived autonomy. Third, a community-based survey with 298 valid participants examined the moderated mediation model in real-world smart older adult care settings.

**Results:**

In study 1, compared with the low-intensity condition, the high-intensity condition significantly increased decisional substitution and perceived surveillance, while reducing perceived autonomy. Indirect effects through decisional substitution (effect=−0.072, 95% CI −0.144 to −0.016) and perceived surveillance (effect=−0.084, 95% CI −0.161 to −0.019) were both significant. In study 2, perceived algorithmic care intensity positively predicted decisional substitution (β=.400, *P*<.001) and perceived surveillance (β=.440, *P*<.001), and negatively predicted perceived autonomy (β=−.356, *P*<.001). The indirect effects through decisional substitution (effect=−0.112, 95% CI −0.172 to −0.065) and perceived surveillance (effect=−0.149, 95% CI −0.215 to −0.093) were significant. Digital literacy significantly weakened the effect of algorithmic care intensity on decisional substitution (β=−.174, *P*=.002), but not on perceived surveillance (β=−.071, *P*=.18).

**Conclusions:**

Algorithmic care may undermine older adults’ perceived autonomy both directly and indirectly through decisional substitution and perceived surveillance. Digital literacy appears to be a selective rather than universal buffer. These findings extend smart older adult care research beyond technology acceptance and clarify the tension between empowerment and control in algorithmic care.

## Introduction

### Smart Older Adult Care and the Emerging Autonomy Question

As population aging deepens and digital technologies become increasingly embedded in community care, smart older adult care is shifting from basic information support to more continuous, algorithm-driven care [1]. Wearable devices, home sensor systems, remote health platforms, and intelligent alert tools are now widely used for fall detection, chronic disease management, risk identification, and daily support. These technologies can ease pressure on care resources, improve response efficiency, and support aging in place [2]. At the same time, as data collection, condition recognition, and automated reminders become part of older adults’ daily lives, smart older adult care also raises concerns about excessive monitoring, technological dependence, privacy exposure, and weakened care relationships [2-5]. The key issue is therefore no longer only whether such technologies are usable, but whether they change how older adults experience themselves as active subjects in care [6].

Existing research has examined smart older adult care and digital health technologies mainly through technology adoption, usage intention, functional performance, and implementation barriers. Previous studies show that perceived usefulness, ease of use, digital literacy, external support, organizational resources, deployment conditions, and service fit shape older adults’ acceptance and use of digital technologies [7,8]. Other studies emphasize the benefits of smart monitoring and digital care tools for health management, risk warning, and continuity of care [9,10]. Although this literature has advanced understanding of smart older adult care, its main focus remains whether older adults are willing and able to use technology. Less attention has been paid to a more psychological question: when care increasingly relies on system recommendations, automated reminders, and continuous monitoring, do older adults still feel that they are directing their own lives, or do they begin to feel managed by the system [11]?

### From Technology Adoption to Psychological Mechanisms

Research grounded in self-determination theory suggests that autonomy, competence, and relatedness are central to older adults’ engagement with digital health technologies. Technology is more likely to support independent living when it preserves choice, navigation, and personalized control [12]. At the same time, studies of monitoring technologies show that older adults’ concerns extend beyond informational privacy to dignity, control, and the feeling of being watched [13]. Research on intelligent care systems similarly suggests that technologies designed to improve efficiency and safety may also strengthen discipline, relational distance, and external control [14]. However, these concerns are often discussed separately. Existing research has not yet provided a clear model explaining how algorithmic care may undermine older adults’ perceived autonomy and through which psychological pathways this occurs. Nor has it adequately examined whether these effects vary according to older adults’ digital capabilities.

This study responds to these gaps by moving the discussion of community smart older adult care beyond technology adoption and toward perceived autonomy. It examines how perceived algorithmic care intensity influences older adults’ perceived autonomy through 2 parallel psychological pathways, decisional substitution and perceived surveillance, and whether digital literacy moderates these effects. Decisional substitution refers to the feeling that care arrangements, health judgments, and everyday decisions are increasingly preset, guided, or replaced by the system, whereas perceived surveillance refers to the sense that one’s behavior, physical condition, and daily routines are continuously observed and recorded by the technological system. The former reflects weakened control over action and decision making, whereas the latter reflects disruption of psychological boundaries.

### This Study and Its Contributions

In this study, perceived algorithmic care intensity does not refer to a single device category or fixed technical function. Rather, it captures older adults’ overall perception of how deeply algorithmic systems are involved in the surrounding care environment, especially through proactive system participation, automated evaluative input, and system-shaped follow-up arrangements. On this basis, the study develops a parallel mediation framework with first-stage moderation to examine a central tension in smart older adult care—whether algorithmic care supports independent living or instead weakens older adults’ agency and control in the name of safety and efficiency.

This study makes 3 main contributions. First, it extends smart older adult care research beyond technology adoption by showing how algorithmic care shapes older adults’ perceived autonomy. Second, it identifies decisional substitution and perceived surveillance as 2 distinct psychological pathways through which algorithmic care affects autonomy. Third, it shows that digital literacy functions as a selective boundary condition, buffering decisional substitution more than perceived surveillance, thereby offering a more refined basis for the design of human-centered community smart older adult care.

### Literature Review

#### Algorithmic Care and Older Adults’ Perceived Autonomy

Hypothesis 1: The higher the perceived intensity of algorithmic care, the lower older adults’ perceived autonomy.

According to self-determination theory, autonomy does not mean acting without external support or completing all activities independently [15]. Rather, it refers to the subjective experience that one’s actions are voluntary, self-endorsed, and guided by personal choice [16,17]. In community smart older adult care, perceived autonomy refers to older adults’ perceived ability to remain the primary agents of everyday care-related arrangements, daily rhythms, and responses to health and living needs while using smart care services. It therefore concerns care-related everyday autonomy within technology-supported community living, rather than formal medical autonomy over clinical treatment or legal data autonomy over personal information governance.

As people age, physical decline, chronic disease management, and increasing dependence on care can make daily life more shaped by family members, community services, and institutional arrangements [18-20]. In this context, perceived autonomy is closely linked to dignity, self-worth, life continuity, and the ability to maintain an ordinary life on one’s own terms [21]. Research further suggests that positive experiences with digital health systems depend not only on functional convenience, but also on whether technology supports autonomy, competence, and social connection [12]. When systems are easy to navigate, interactive, and customizable, older adults are more likely to view them as resources for self-management rather than sources of external control [22].

However, algorithmic care creates new challenges for perceived autonomy. Unlike traditional care, it increasingly intervenes in risk detection, health assessment, anomaly alerts, behavioral reminders, and daily management [23]. In older adult care and digital monitoring settings, such technologies may improve safety and efficiency while also intensifying concerns about control, dignity, constant observation, discipline, relational distance, and external control [14]. Thus, the more strongly older adults perceive algorithmic care to shape daily care, health management, and life arrangements through system recognition, automated judgment, and system-initiated follow-up, the more likely they are to experience reduced control and choice in the care process.

#### Algorithmic Care and Decisional Substitution

Hypothesis 2: The higher the perceived intensity of algorithmic care, the stronger older adults’ sense of decisional substitution.

In community smart older adult care, algorithmic care deserves attention not only because it can improve efficiency, but also because it may reshape older adults’ role in decision-making [11]. Decisional substitution refers to the subjective feeling that matters once requiring one’s own judgment, weighing, and choice are increasingly preset, guided, or handled by an external system [24,25]. Rather than treating technology as a simple source of information support, this concept captures a psychological experience in which one’s room for participation, judgment, and control is reduced [26,27]. Research on shared decision-making shows that active participation in decisions is central to maintaining agency and self-direction; when decisions are shaped mainly by external authority, procedural rules, or technical logic, individuals are more likely to shift from active decision makers to passive recipients [16].

Algorithmic care increasingly performs functions such as risk detection, anomaly alerts, health assessment, behavioral reminders, and task prioritization [28]. As such systems move from supportive tools to active participants in judgment, older adults may feel that important decisions are no longer truly made by themselves but are prestructured or partly replaced by system logic [29]. Recent studies have therefore emphasized deliberation, customization, and human-centered loops in preserving users’ decision-making role [30]. Without adequate room for participation, excessive reliance on automation and monitoring may create tension between supporting care and replacing the care recipient’s own judgment [31].

From this perspective, higher perceived algorithmic care intensity implies not only deeper technological involvement, but also a shift in judgmental authority. The more older adults feel that care, health management, and daily arrangements depend on system recognition, automated triggers, and programmed recommendations, the more their space for self-judgment and initiative may be reduced. As a result, they are more likely to develop a stronger sense of decisional substitution.

#### Algorithmic Care and Perceived Surveillance

Hypothesis 3: The higher the perceived intensity of algorithmic care, the stronger older adults’ perceived surveillance.

Beyond decisional substitution, perceived surveillance represents another key pathway through which algorithmic care may shape psychological outcomes. Perceived surveillance refers to the subjective feeling that one’s behavior, physical condition, and daily routines are continuously observed, recorded, and analyzed by a technological system [3,32]. Unlike ordinary information collection, it captures a situated experience in which individuals feel that their lives are being constantly seen, interpreted, and even anticipated. Previous studies suggest that in health monitoring and assisted living settings, users’ concerns extend beyond data leakage to dignity, control, autonomy, and the experience of intrusive observation [4,33-35].

This logic is especially salient in community smart older adult care. Traditional care relies mainly on face-to-face observation and periodic assessment, whereas algorithmic care is typically built on continuous sensing, anomaly detection, and real-time feedback [36]. Wearable devices and home sensor systems transform physiological states, movement patterns, daily routines, and unusual behaviors into data that can be tracked, recorded, and evaluated [37]. Although such systems may improve risk warning and service response, they may also blur the boundary between supportive care and constant observation [38].

This risk arises not simply because information is collected, but because technology turns everyday life into something continuously visible, recordable, and interpretable. As monitoring devices intensify this visibility, older adults may evaluate technology not only in terms of safety, but also in terms of whether it leaves them feeling constantly seen [39]. Perceived surveillance may therefore reshape how power is experienced within the care relationship [40], especially when smart technologies become deeply embedded in care institutions and daily routines [14]. Thus, the more older adults feel that their health status, activity patterns, and daily arrangements depend on continuous data capture and automated recognition, the more likely they are to feel watched rather than merely supported [41].

#### The Mediating Roles of Decisional Substitution and Perceived Surveillance in Perceived Autonomy

Hypothesis 4a: Decisional substitution mediates the relationship between perceived algorithmic care intensity and older adults’ perceived autonomy.

Hypothesis 4b: Perceived surveillance mediates the relationship between perceived algorithmic care intensity and older adults’ perceived autonomy.

Although algorithmic care may provide timely alerts, support continuity in health management, and reduce care uncertainty, its implications extend beyond convenience and safety. As argued in previous sections, stronger algorithmic care may heighten both decisional substitution and perceived surveillance. Self-determination theory suggests that autonomy depends not on complete independence from external support, but on whether individuals feel that their actions reflect their own will and that important choices are made through understanding, participation, and endorsement [16,17]. When an external system repeatedly narrows a person’s space for judgment, choice, and interpretation, perceived autonomy is likely to decline even if the system is intended to protect, assist, or optimize [3,33].

Decisional substitution is likely to reduce perceived autonomy because it weakens the sense that older adults remain active participants in directing their own lives [42]. Research on shared decision-making shows that involvement in understanding information, weighing options, and selecting outcomes is essential for maintaining agency and self-direction [43]. When key matters are preset, prioritized, or automatically triggered by an external system, individuals are more likely to move from active participants to passive executors [44,45]. In community smart older adult care, this shift may emerge as older adults become accustomed to relying on system reminders, assessments, and generated priorities [46]. Over time, when the system judges first and the individual responds afterward, the experience of self-directed action is likely to weaken [29]. Related research in virtual care and long-term care similarly suggests that technology-mediated care can narrow deliberative space, which is an early sign of declining perceived autonomy [47].

Perceived surveillance is likely to reduce perceived autonomy through a different mechanism: the disruption of psychological boundaries. When bodily states, movement patterns, and daily routines are continuously observed, recorded, and analyzed, the experience is no longer simply one of information collection, but of being constantly seen and interpreted [32,33]. This ongoing visibility reshapes private space, behavioral freedom, and self-control [4]. Previous research shows that resistance to monitoring technologies often centers on privacy, intrusiveness, stigma, and the feeling of being watched [48]. When technology conflicts with users’ values or everyday practices, systems intended to support independent living may instead be experienced as intrusions into personal boundaries and daily rhythms [5]. In this sense, perceived surveillance is not only a privacy concern, but also a challenge to whether one can still live freely in one’s own way.

#### The Moderating Role of Digital Literacy and Conditional Indirect Effects

Hypothesis 5a: Digital literacy moderates the relationship between algorithmic care intensity and decisional substitution, such that this relationship is stronger for older adults with lower digital literacy than for those with higher digital literacy.

Hypothesis 5b: Digital literacy moderates the relationship between algorithmic care intensity and perceived surveillance, such that this relationship is stronger for older adults with lower digital literacy than for those with higher digital literacy.

The preceding arguments suggest that algorithmic care intensity affects perceived autonomy through decisional substitution and perceived surveillance. However, these mechanisms may not operate uniformly across individuals. Older adults differ in how well they understand digital systems, how confidently they use them, and how they interpret system outputs. Digital literacy has been widely recognized as a key factor shaping older adults’ experiences with and evaluations of technology [49]. Older adults with higher digital literacy are generally better able to understand how digital systems work, interpret system outputs, and use technology with confidence, whereas lower digital literacy is associated with greater difficulty interpreting digital information and engaging with technology-based services [50,51]. Because digital literacy reflects not only tool use but also basic understanding of system operation and data processing [52], it may serve as an important boundary condition in responses to algorithmic care.

Previous research suggests that individuals with higher digital literacy are more likely to view technological systems as instrumental support rather than as substitutes for their own control, and are therefore better able to maintain a sense of agency when using them [51,53]. In smart older adult care settings, stronger algorithmic care may generate reminders, warnings, and recommended courses of action. For older adults with higher digital literacy, such interventions are more likely to be interpreted as supportive suggestions because they are better able to understand system logic and evaluate system outputs [54]. For those with lower digital literacy, however, frequent prompts and automated judgments may be experienced as a transfer of decision-making authority, thereby strengthening decisional substitution.

Digital literacy may also shape interpretations of monitoring, because older adults with stronger technical understanding may be better able to distinguish data recording from social surveillance [55]. Therefore, higher digital literacy may weaken the association between algorithmic care intensity and perceived surveillance.

**Figure 1.**
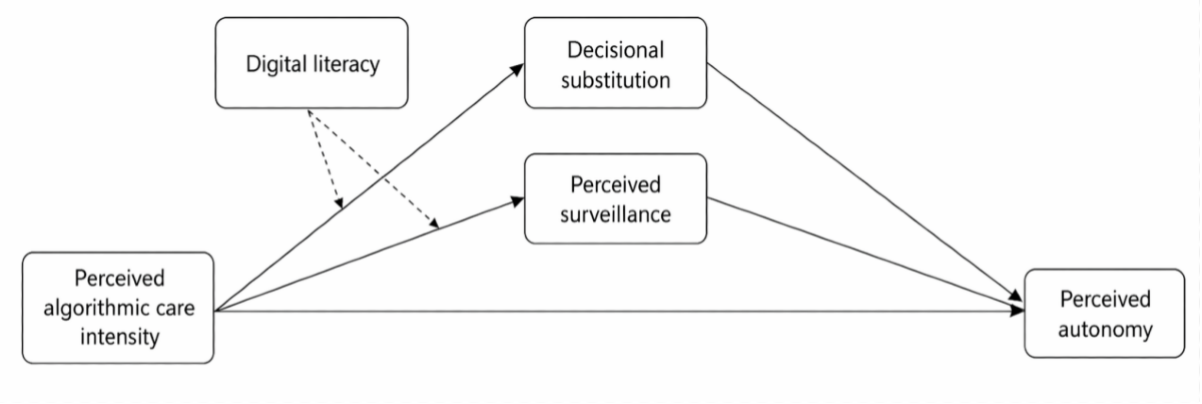
Research model.

The research model is illustrated in Figure 1.

## Methods

### Overview

This study adopted a multistage design combining a qualitative prestudy, a vignette-based experiment, and a field survey of older adults in real community settings. This design was used to improve contextual validity, establish causal direction, and test the proposed model in real-world smart older adult care contexts. Previous work shows that older adults’ experiences of digital health and smart care technologies are shaped by everyday contexts, especially in relation to autonomy, privacy, surveillance, and control [12,56]. Reviews of monitoring-based assisted living technologies have also noted limitations related to ecological validity, end user orientation, and weak identification of underlying mechanisms [32,48]. Accordingly, we first used semistructured interviews to refine measurement items and vignette materials, then conducted an experiment to test causal effects, and finally validated the model using a community-based survey. In the main studies, participants were recruited in participating communities by the author and the author’s student research team, who also assisted with on-site study administration.

### Prestudy: Semistructured Interviews and Measure Development

Before the main studies, we conducted semistructured interviews to examine whether older adults described community smart older adult care in terms such as “things are no longer decided by me,” “the system makes judgments first,” or “I feel watched.” The goal was not to test hypotheses, but to strengthen content validity and contextual fit by incorporating older adults’ own language and everyday experiences [12,56].

Using purposive sampling, we recruited 15 community residents aged 60 years or older from an older adult care community in Hangzhou, China. All participants had used or been exposed to community smart older adult care services or related devices within the past year. To increase heterogeneity, we sought variation in age, education, living arrangement, chronic disease status, and prior experience with digital technologies. Interviews followed a semistructured guide covering system involvement, perceived loss of decision control, continuous recording or observation, and perceived autonomy, comfort, control, and respect. Each interview lasted about 30-50 minutes. With consent, interviews were audio recorded and transcribed.

The transcripts were analyzed using thematic analysis. Moreover, 2 members of the research team independently reviewed the transcripts, conducted open coding, compared coding results, and resolved differences through iterative discussion until agreement was reached on higher-order themes. The resulting themes were used to revise item wording and inform the vignette materials for study 1, thereby reducing the gap between researchers’ conceptual assumptions and older adults’ lived experiences [57].

### Study 1: Vignette-Based Experiment

Study 1 tested whether perceived algorithmic care intensity affects decisional substitution, perceived surveillance, and perceived autonomy. A vignette-based experiment was used because cross-sectional survey data cannot rule out reverse causality, and older adults’ responses to monitoring and digital care technologies are strongly shaped by specific use scenarios [13,56].

Study 1 used a 1-factor, 2-level between-subjects design. The independent variable was algorithmic care intensity, manipulated as a high intensity versus low intensity scenario. In the high intensity condition, the community smart older adult care system continuously collected information about older adults’ activities, routines, and health status, automatically generated reminders, risk alerts, and care suggestions, and used these outputs to shape subsequent support and care arrangements. In the low intensity condition, the system mainly provided basic recording and information display, while subsequent support and care arrangements relied primarily on discussion between the older adult and community staff. In both conditions, community staff remained present as part of the care context. The 2 vignettes were matched in length, writing style, and background context.

We recruited 255 older adults aged 60 years or older from 2 large communities in Hangzhou, China, and randomly assigned them to one of the 2 conditions. Participants first received a brief explanation of the study and provided informed consent. They were then presented with 1 vignette at random and completed manipulation check items and the main measures. The manipulation check assessed whether participants perceived the system as highly reliant on recognition, automated reminders, and programmed judgment. The main measures assessed perceived algorithmic care intensity, decisional substitution, perceived surveillance, and perceived autonomy. Age, gender, education, self-rated health, and previous experience with digital technologies were also collected as control variables.

### Study 2: Community-Based Field Survey

Study 2 examined whether the proposed model holds in real community smart older adult care settings and whether digital literacy moderates the proposed mechanisms. Because older adults’ evaluations of smart care technologies depend on actual environments, interaction patterns, and the extent to which technology is embedded in daily life, vignettes alone cannot demonstrate whether the model applies to real use contexts [12].

Participants were recruited through on-site distribution with researcher-assisted completion in 3 large communities, day care centers, and community health service stations in Hangzhou and Ningbo, China. These 2 cities were selected because community smart older adult care services are relatively well developed there. A total of 330 older adults participated. Eligibility criteria were (1) aged 60 years or older; (2) having used or been exposed to at least 1 type of community smart older adult care service in the past year, such as a health monitoring system, remote consultation platform, smart emergency call device, or data management platform; and (3) having basic reading ability and being able to complete the questionnaire with assistance if needed. Before the survey, researchers provided a standardized explanation of “smart older adult care services” and instructed participants to answer based on their overall experience of algorithmic involvement across the services they had used, rather than focusing on a single device or application.

Study 2 measured the same core constructs as study 1, that is, perceived algorithmic care intensity, decisional substitution, perceived surveillance, and perceived autonomy. Digital literacy was measured as the moderator. To reduce common method bias, the questionnaire visually separated different constructs into sections and included filler items to weaken consistency responding.

We estimated a moderated mediation model to test whether digital literacy attenuates the positive effect of algorithmic care intensity on decisional substitution and perceived surveillance. Multiple regression and bias-corrected bootstrap procedures were used to estimate conditional indirect effects, and moderation was tested through interaction terms. Age, gender, and education were included as control variables in the main regression analyses.

### Statistical Analysis and Measures

This study measured five core constructs: (1) perceived algorithmic care intensity [13,56], (2) decisional substitution [12,13], (3) perceived surveillance [32,48], (4) perceived autonomy [22], and (5) digital literacy [58]. Because older adults’ experiences with digital health and smart care technologies are highly contextual and subjective, the items were adapted from previous studies and refined through the prestudy interviews to fit community smart older adult care settings [12,56].

All formal measures used a 7-point Likert scale, ranging from 1=strongly disagree to 7=strongly agree. Study 1 and study 2 followed the same general measurement framework, with minor wording adjustments to fit the research context. In study 1, items referred to “the community smart older adult care system described above,” whereas in study 2, they referred to “the community smart older adult care services you are currently using.” This approach helped maintain construct consistency while improving contextual relevance and clarity.

Perceived algorithmic care intensity measured the extent to which older adults felt that the surrounding care process relied on system recognition, automated reminders, programmed recommendations, backend judgment, and technology-shaped follow-up arrangements. The construct captured older adults’ higher-order perception of how deeply algorithmic systems were embedded in the care environment, rather than any single device type, alert frequency, or task criticality [13,56].

Decisional substitution measured the extent to which older adults felt that matters requiring their own judgment and choice were instead preset, guided, or handled by the system. This measure was grounded in self-determination theory and refined with reference to research on shared decision making and user participation [12,13].

Perceived surveillance measured the subjective feeling that one’s behavior, physical condition, and daily routines were continuously observed, recorded, and analyzed by the technological system. This measure was informed by research on monitoring technologies, camera-based assisted living, and digital care systems for older adults [32,48].

Perceived autonomy captured whether older adults still felt that they remained the primary agents of everyday care-related arrangements, daily routines, and responses to health and living needs in community smart older adult care. The construct focused on agency, choice, and behavioral self-direction in ordinary technology-supported care, rather than formal medical decision autonomy or legal data autonomy [22].

Digital literacy measured older adults’ ability to understand, access, and use digital technologies and digital health information [58]. A shortened digital literacy scale was adopted for this purpose.

In addition to the focal constructs, study 2 collected background information on age, gender, education level, marital status, self-rated health, number of chronic conditions, living arrangement, and frequency of smart older adult care service use. Age, gender, and education were included as controls in the reported regression analyses.

### Ethical Considerations

This study received ethical approval from the institutional ethics committee of the authors’ affiliated university (approval 2025-HUM-021) and was conducted in accordance with the Declaration of Helsinki. Data were collected between March and June 2025 from adults aged 60 years and older, and no minors were involved. All participants provided informed consent before participation. Participation was voluntary, and all responses were collected anonymously and used solely for academic research purposes. This study was not registered in a clinical trial registry because it was not designed as a prospective clinical intervention trial. Instead, it used a 3-stage design combining a qualitative prestudy, a vignette-based experiment, and a community-based field survey.

## Results

### Prestudy: Semistructured Interviews and Measure Development

The qualitative prestudy confirmed that the focal constructs were meaningful and recognizable in community smart older adult care settings. A total of 15 older adults who had used or been exposed to community smart older adult care services participated in semistructured interviews. Detailed participant characteristics are reported in Multimedia Appendix 1. The interviews examined how participants understood system involvement, loss of decision control, continuous recording or observation, and perceived autonomy, comfort, control, and respect. The purpose was not to test hypotheses, but to generate empirical input for item refinement and vignette development [12,56].

The thematic analysis yielded 6 representative themes, which are summarized in Multimedia Appendix 1. Overall, participants did not evaluate smart older adult care only in terms of convenience or technological advancement. Instead, they repeatedly focused on whether algorithm-enabled involvement changed their position in the care process. Moreover, 3 patterns were especially salient. First, participants recognized safety and convenience benefits, especially for alerts, health monitoring, chronic disease management, and reducing burdensome medical visits. Second, they became more sensitive when the system moved from information display to active judgment, reminders, and arrangement shaping, which was associated with a perceived weakening of their own decision-making role. Third, continuous monitoring was often experienced not simply as data collection, but as ongoing visibility, boundary disruption, and reduced personal control. Across these accounts, a common concern emerged, that is, whether older adults could still remain the person in charge.

These findings informed both measure refinement and vignette design. Expressions such as “many things are no longer decided by me first” informed decisional substitution items, whereas expressions such as “it feels like there are always eyes in the house” informed perceived surveillance items. Items for perceived algorithmic care intensity emphasized proactive identification, automated reminders, programmed judgments, and system-shaped care arrangements. Items for perceived autonomy focused on whether participants still felt able to decide, retain freedom of choice, and live according to their own rhythm within smart care arrangements. The high-intensity vignette emphasized continuous monitoring, automated alerts, backend analysis, and system-shaped follow-up arrangements, whereas the low-intensity vignette emphasized basic recording and information display, with subsequent support relying more on discussion between older adults and community staff.

### Study 1: Results of the Vignette-Based Experiment

#### Overview

After the prestudy confirmed that the focal constructs fit the smart older adult care context and after the measures and vignette materials were refined, we conducted study 1 to test the effects of perceived algorithmic care intensity on decisional substitution, perceived surveillance, and perceived autonomy. A total of 255 older adults were recruited. Responses with substantial missing data on the main study variables were excluded before analysis. As a result, 233 valid responses were retained for analysis, including 115 participants in the low algorithmic care intensity group and 118 in the high algorithmic care intensity group. The final analyses were conducted on complete cases, and no statistical imputation procedure was applied. The mean age of the sample was 69.70 (SD 6.24) years, and 127 out of 233 (54.5%) participants were female. Randomization checks showed no significant between-group differences in age, number of chronic conditions, gender, education, living arrangement, or digital literacy (all *P* values>.05), suggesting that random assignment was effective and that the 2 groups were comparable.

#### Manipulation Check

To examine whether the experimental manipulation was successful, we first compared the 2 groups on the manipulation check scale. The results showed that the high algorithmic care intensity group reported significantly higher perceived algorithmic care intensity than the low intensity group. Specifically, the mean score was 4.79 (SD 0.95) in the high intensity condition and 4.11 (SD 0.91) in the low intensity condition, and the difference was significant (2-tailed *t*_231_=5.63; Cohen *d*=0.74; *P*<.001).

#### Reliability, Descriptive Statistics, and Correlation Analysis

Before testing the hypotheses, we examined the internal consistency of all measures. The results showed acceptable reliability for all scales: Cronbach α was 0.753 for the manipulation check scale, 0.695 for decisional substitution, 0.716 for perceived surveillance, and 0.782 for perceived autonomy. Although the α for decisional substitution was close to the conventional threshold, it remained within an acceptable range for a brief vignette-based measure, and the scale was retained because it captured the focal construct with adequate conceptual coverage in the study 1 context.

Tables1  2 present the means, SDs, reliabilities, and correlations among the variables. Perceived algorithmic care intensity was positively correlated with decisional substitution (*r*=0.27, *P*<.001) and perceived surveillance (*r*=0.25, *P*<.001), and negatively correlated with perceived autonomy (*r*=−0.22, *P*<.001). In addition, decisional substitution was negatively correlated with perceived autonomy (*r*=−0.25, *P*<.001), and perceived surveillance was also negatively correlated with perceived autonomy (*r*=−0.29, *P*<.001). Notably, decisional substitution and perceived surveillance were positively related, but the correlation was not significant (*r*=0.05, *P*=.41). This suggests that although both reflect negative psychological responses to algorithmic care, they do not fully overlap in meaning. This pattern also supports treating them as 2 parallel mediators.

**Table 1.** Descriptive statistics and reliability for study 1 variables.

Variable	Mean (SD)	Cronbach α
Perceived algorithmic care intensity	4.45 (0.99)	0.753
Decisional substitution	4.28 (0.90)	0.695
Perceived surveillance	4.11 (0.93)	0.716
Perceived autonomy	3.86 (0.99)	0.782

**Table 2.** Correlation analysis for study 1 variables.

Variable	Perceived algorithmic care intensity	Decisional substitution	Perceived surveillance	Perceived autonomy
Perceived algorithmic care intensity				
*r*	1	0.27	0.25	−0.22
*P* value	—^a^	<.001	<.001	<.001
Decisional substitution				
*r*	0.27	1	0.05	−0.25
*P* value	<.001	—	.41	<.001
Perceived surveillance				
*r*	0.25	0.05	1	−0.29
*P* value	<.001	.41	—	<.001
Perceived autonomy				
*r*	−0.22	−0.25	−0.29	1
*P* value	<.001	<.001	<.001	—

a Not applicable.

#### Hypothesis Testing

To test hypotheses 1-3, we used experimental condition (low algorithmic care intensity=0, high algorithmic care intensity=1) as the independent variable and compared the 2 groups on decisional substitution, perceived surveillance, and perceived autonomy. As presented in Tables3  4, compared with the low intensity condition, the high algorithmic care intensity condition significantly increased decisional substitution (mean 4.43, SD 0.94 vs mean 4.13, SD 0.84; *t*_231_=2.64; Cohen *d*=0.35; *P*=.009) and perceived surveillance (mean 4.27, SD 0.91 vs mean 3.95, SD 0.95, *t*_231_=2.58; Cohen *d*=0.34; *P*=.01), while significantly reducing perceived autonomy (mean 3.66, SD 1.03 vs mean 4.06, SD 0.91, *t*_231_=−3.17; Cohen *d*=−0.42; *P*=.002). These results support hypotheses 1-3, indicating that stronger algorithmic care increased decisional substitution and perceived surveillance while reducing perceived autonomy.

**Table 3.** Manipulation check and hypothesis testing results.

Variable	Low algorithmic care intensity, mean (SD)	High algorithmic care intensity, mean (SD)	*t* test (*df*)	Cohen *d*	*P* value	Conclusion
Perceived algorithmic care intensity (manipulation check)	4.11 (0.91)	4.79 (0.95)	5.63 (231)	0.74	<.001	Manipulation successful
Decisional substitution	4.13 (0.84)	4.43 (0.94)	2.64 (231)	0.35	.009	H2 supported
Perceived surveillance	3.95 (0.95)	4.27 (0.91)	2.58 (231)	0.34	.01	H3 supported
Perceived autonomy	4.06 (0.91)	3.66 (1.03)	−3.17 (231)	−0.42	.002	H1 supported

**Table 4.** Bootstrap results for the parallel mediation effects in study 1.

Path	Effect (95% CI)	Conclusion
Algorithmic care intensity → decisional substitution → perceived autonomy	−0.072 (−0.144 to −0.016)	H4a supported
Algorithmic care intensity → perceived surveillance → perceived autonomy	−0.084 (−0.161 to −0.019)	H4b supported
Total indirect effect	−0.156 (−0.262 to −0.066)	Significant
Direct effect	−0.247 (−0.491 to −0.006)	Still significant, partial mediation
Total effect	−0.403 (−0.650 to −0.153)	Significant

We next tested the parallel mediating roles of decisional substitution and perceived surveillance using bootstrap analysis with 5000 resamples. Experimental condition was entered as the independent variable, perceived autonomy as the dependent variable, and decisional substitution and perceived surveillance as parallel mediators. As illustrated in Tables3  4, the indirect effect through decisional substitution was significant (effect=−0.072, 95% CI −0.144 to −0.016), supporting hypothesis 4a. The indirect effect through perceived surveillance was also significant (effect=−0.084, 95% CI −0.161 to −0.019), supporting hypothesis 4b. The total indirect effect was −0.156 (95% CI −0.262 to −0.066). After both mediators were included, the direct effect of algorithmic care intensity on perceived autonomy remained significant (effect=−0.247, 95% CI −0.491 to −0.006), indicating partial mediation.

### Study 2: Results of the Community-Based Field Survey

#### Overview

Study 2 was designed to further test the applicability and robustness of the proposed model in real community smart older adult care settings and to examine the moderating role of digital literacy in the first-stage paths. Given that participants had experience with different smart older care services and devices, the findings should be interpreted as reflecting the perceived psychological consequences of algorithmic care in a heterogeneous real-world service environment rather than the effects of any single technology category.

A total of 298 valid responses were retained for analysis. Responses with substantial missing data on the focal variables were excluded before analysis. The final analyses were conducted on complete cases, and no statistical imputation procedure was applied. The mean age of the participants was 71.8 (SD 6.202) years, and 158 out of 298 (53%) participants were female. In terms of education, 221 out of 298 (74%) participants had completed high school or below, whereas 77 out of 298 (26%) participants had completed junior college or above. Although most participants had completed high school or below, the proportion with junior college education or above and the urban locations of Hangzhou and Ningbo suggest that the sample reflects older adults in relatively developed community smart care settings. This context is substantively appropriate for examining the focal mechanisms of algorithmic care, although the strength and form of these relationships may vary across regions with different levels of digital development and care infrastructure.

#### Measurement Model

Before hypothesis testing, we examined the descriptive statistics and correlations among the focal variables. Perceived algorithmic care intensity was positively correlated with decisional substitution and perceived surveillance, and negatively correlated with perceived autonomy. Decisional substitution and perceived surveillance were also positively related to each other and both were negatively associated with perceived autonomy.

Because study 2 relied on self-reported questionnaire data, we also assessed the potential risk of common method bias. In addition to the procedural remedies applied during questionnaire design, we conducted Harman’s single factor test and compared the proposed 5-factor model with a single factor model. The results indicated that common method bias was unlikely to materially distort the measurement structure.

We next assessed the reliability and validity of the measures. All Cronbach α values exceeded the conventional threshold of 0.70, indicating strong internal consistency. Confirmatory factor analysis showed that the 5-factor measurement model fit the data well, with *χ*²_179_=353.88, comparative fit index=0.947, Tucker-Lewis index=0.938, root-mean-square error of approximation=0.057, and standardized root-mean-square residual=0.049, supporting the structural distinctiveness of perceived algorithmic care intensity, decisional substitution, perceived surveillance, perceived autonomy, and digital literacy. In addition, all standardized factor loadings exceeded 0.80, and both composite reliability and average variance extracted were above recommended thresholds, supporting convergent validity. Detailed results are reported in Table 5.

To further assess discriminant validity, we used the heterotrait-monotrait ratio (HTMT). As illustrated in Table 6, all HTMT values were below 0.85, indicating adequate discriminant validity across constructs.

**Table 5.** Measurement model results for study 2.

Constructs	Number of items	Factor loading range	CR^a^	AVE^b^	Cronbach α
Perceived algorithmic care intensity	3	0.812‐0.895	0.919	0.791	0.920
Decisional substitution	4	0.838‐0.877	0.919	0.740	0.918
Perceived surveillance	4	0.840‐0.868	0.916	0.732	0.915
Perceived autonomy	4	0.852‐0.882	0.923	0.750	0.922
Digital literacy	6	0.841‐0.901	0.954	0.776	0.954

a CR: composite reliability.

b AVE: average variance extracted.

**Table 6.** Heterotrait-monotrait ratio results for discriminant validity in study 2.

Variable	Perceived algorithmic care intensity	Decisional substitution	Perceived surveillance	Perceived autonomy	Digital literacy
Perceived algorithmic care intensity					
*r*	1	0.389	0.445	−0.356	0.065
*P* value	—^a^	<.001	<.001	<.001	.27
Decisional substitution					
*r*	0.389	1	0.285	−0.425	0.085
*P* value	<.001	—	<.001	<.001	.14
Perceived surveillance					
*r*	0.445	0.285	1	−0.463	0.037
*P* value	<.001	<.001	—	<.001	.52
Perceived autonomy					
*r*	−0.356	−0.425	−0.463	1	0.045
*P* value	<.001	<.001	<.001	—	.44
Digital literacy					
*r*	0.065	0.085	0.037	0.045	1
*P* value	.27	.14	.52	.44	—

a Not applicable.

#### Tests of Main and Mediating Effects

After confirming the measurement model, we tested the main effects and mediation effects. Consistent with the reported regression specification, age, gender, and education were included as controls in these models. As presented in Table 7, controlling for these variables, perceived algorithmic care intensity significantly and positively predicted decisional substitution (β=.400, SE 0.053; *t*_293_=7.550; *P*<.001) and perceived surveillance (β=.440, SE 0.053; *t*_293_=8.250; *P*<.001), and significantly and negatively predicted perceived autonomy (β=−.356, SE 0.053; *t*_293_=−6.501; *P*<.001), supporting hypotheses 1-3. When decisional substitution and perceived surveillance were entered simultaneously, both negatively predicted perceived autonomy (β=−.294, SE 0.053; *t*_291_=−5.533; *P*<.001; β=−.344, SE 0.055; *t*_291_=−6.287; *P*<.001), whereas the direct effect of perceived algorithmic care intensity became nonsignificant (β=−.087, SE 0.055; *t*_291_=−1.526; *P*=.13), indicating that the effect operated mainly through the 2 mediators. As presented in Table 8, bootstrap analysis further showed significant indirect effects through decisional substitution (effect=−0.112, 95% CI −0.172 to −0.065) and perceived surveillance (effect=−0.149, 95% CI −0.215 to −0.093). The total indirect effect was −0.261 (95% CI −0.346 to −0.189), whereas the direct effect was not significant (effect=−0.085, 95% CI −0.185 to 0.025), indicating full mediation.

**Table 7.** Regression results for main and mediating effects in study 2.

Dependent variable	Independent variable	β (SE)	*t* test (*df*)	*P* value	*R*²
Decisional substitution	Perceived algorithmic care intensity	.400 (0.053)	7.550 (293)	<.001	0.193
Perceived surveillance	Perceived algorithmic care intensity	.440 (0.053)	8.250 (293)	<.001	0.217
Perceived autonomy (total effect)	Perceived algorithmic care intensity	−.356 (0.053)	−6.501 (293)	<.001	0.130
Perceived autonomy (with mediators)	Perceived algorithmic care intensity	−.087 (0.055)	−1.526 (291)	.13	0.319
Perceived autonomy (with mediators)	Decisional substitution	−.294 (0.053)	−5.533 (291)	<.001	—^a^
Perceived autonomy (with mediators)	Perceived surveillance	−.344 (0.055)	−6.287 (291)	<.001	—

a Not applicable.

**Table 8.** Bootstrap results for the parallel mediation effects in study 2.

Path	Effect (95% CI)	Conclusion
Perceived algorithmic care intensity → decisional substitution → perceived autonomy	−0.112 (−0.172 to −0.065)	H4a supported
Perceived algorithmic care intensity → perceived surveillance → perceived autonomy	−0.149 (−0.215 to −0.093)	H4b supported
Total indirect effect	−0.261 (−0.346 to −0.189)	Significant
Direct effect	−0.085 (−0.185 to 0.025)	Not significant
Total effect	−0.346 (−0.446 to −0.246)	Significant

#### Test of the Moderating Role of Digital Literacy

After obtaining support for the main model, we further examined whether digital literacy moderated the effects of algorithmic care intensity on the 2 first-stage paths. To reduce multicollinearity, both perceived algorithmic care intensity and digital literacy were mean-centered before constructing the interaction terms. We then estimated regression models with decisional substitution and perceived surveillance as dependent variables, including the main effects, the interaction term, and the same control variables used in the main effect models, namely age, gender, and education.

As presented in Table 9, the interaction between perceived algorithmic care intensity and digital literacy was significant for decisional substitution (β=−.174, SE 0.055; *t*_291_=−3.178; *P*=.002), supporting hypothesis 5a. Simple slope analysis showed that the positive effect of algorithmic care intensity on decisional substitution was strongest at low digital literacy (simple slope=0.560), moderate at the mean level (simple slope=0.398), and weakest at high digital literacy (simple slope=0.235). By contrast, the interaction between perceived algorithmic care intensity and digital literacy was not significant for perceived surveillance (β=−.071, SE 0.053; *t*_291_=−1.338; *P*=.18), so hypothesis 5b was not supported. This pattern suggests that digital literacy may buffer the sense that decision making is being replaced by the system, but not sensitivity to continuous monitoring and the accumulation of digital traces.

**Table 9.** Moderating effects of digital literacy in study 2.

Dependent variable and predictor	β (SE)	*t* test (*df*)	*P* value	*R*²
Decisional substitution				0.193
Perceived algorithmic care intensity	.413 (0.051)	7.763 (291)	<.001	
Digital literacy	.075 (0.054)	1.380 (291)	.17	
Algorithmic care intensity×digital literacy	−.174 (0.055)	−3.178 (291)	.002	
Perceived surveillance				0.214
Perceived algorithmic care intensity	.448 (0.051)	8.534 (291)	<.001	
Digital literacy	−.018 (0.053)	−0.339 (291)	.73	
Algorithmic care intensity×digital literacy	−.071 (0.053)	−1.338 (291)	.18	

## Discussion

### Principal Results

This study examined how algorithmic care shapes older adults’ perceived autonomy in community smart older adult care settings. Using a qualitative prestudy, a vignette experiment, and a community-based field survey, we tested whether perceived algorithmic care intensity reduces perceived autonomy through decisional substitution and perceived surveillance, and whether digital literacy moderates these effects.

The results show that algorithmic care is not merely a neutral arrangement for improving efficiency and safety. When care relies more heavily on system recognition, automated reminders, programmed recommendations, and system-shaped follow-up arrangements, older adults are more likely to feel that decisions are no longer made by them first and that their daily lives are continuously recorded and observed. These 2 experiences, in turn, reduce perceived autonomy. This pattern was supported in both the vignette experiment and the community-based field survey.

The findings also show that decisional substitution and perceived surveillance are 2 distinct pathways. Decisional substitution reflects a perceived weakening of older adults’ role in judgment and choice, whereas perceived surveillance reflects the disruption of psychological boundaries caused by continuous observation and data recording. Digital literacy weakened the effect of algorithmic care intensity on decisional substitution, but not on perceived surveillance. This suggests that digital competence may help older adults interpret system outputs as supportive inputs rather than replacements for their own judgment, but it does not necessarily remove discomfort caused by continuous monitoring and digital traces.

### Limitations

This study has several limitations. First, perceived algorithmic care intensity was examined as older adults’ overall perception of algorithmic involvement in the care environment. Different technologies may produce different psychological responses. For example, passive monitoring tools may more directly trigger feelings of being watched, whereas user-initiated advisory applications may allow more decisional participation. Future research could compare passive monitoring systems, user-initiated health applications, and hybrid care platforms.

Second, the study 2 sample was drawn from Hangzhou and Ningbo, where digital infrastructure and smart older adult care implementation are relatively developed. The findings may therefore reflect older adults’ experiences in urban community smart care settings. Future research could test the model in counties, rural areas, and regions with different levels of digital development and care infrastructure.

Third, this study relied mainly on self-report data. Although self-reports are appropriate for capturing subjective experiences such as autonomy, decisional substitution, and perceived surveillance, they do not provide strong behavioral evidence. Future studies could include platform usage logs, alert response records, family intervention frequency, or actual choices made after system recommendations.

Fourth, although this study combined a qualitative prestudy, an experiment, and a field survey, study 2 was still cross-sectional. It therefore could not capture the longer-term adaptation process through which older adults respond to algorithmic care. Future research could use longitudinal or staged designs to examine whether older adults gradually adapt to algorithmic care, become more dependent on it, or develop stronger resistance over time.

### Comparison With Previous Work

Much of the existing smart older adult care literature has focused on technology acceptance, perceived usefulness, usage intention, and implementation barriers [7,8,56]. This work has helped explain whether older adults are willing and able to use digital technologies, but it says less about what happens psychologically after such technologies become embedded in everyday care. Previous work has argued that older adults’ digital health experiences are closely tied to autonomy, competence, and relatedness rather than usefulness alone [12]. The findings of this study extend this view by showing that algorithmic care may support daily living while simultaneously weakening older adults’ sense of self-direction.

This study also extends previous research on monitoring technologies. Existing studies have shown that older adults’ concerns about smart monitoring often go beyond data privacy and include dignity, control, intrusiveness, and the feeling of being watched [33,48]. Research on AI in care settings has similarly noted the tension between efficient care and humane care, especially when automation increases discipline and relational distance [13]. However, these concerns are often discussed together. By distinguishing decisional substitution from perceived surveillance, this study shows that the psychological consequences of algorithmic care are multidimensional. Decisional substitution concerns whether older adults remain part of the process of deciding, whereas perceived surveillance concerns whether they feel constantly seen, recorded, and made legible to the system.

The moderating findings also refine current assumptions about digital inclusion. Policy and practice often assume that improving digital literacy will reduce older adults’ difficulties with smart care technologies. Our results only partly support this view. Digital literacy reduced the likelihood that older adults experienced system outputs as replacing their own judgment, but it did not significantly reduce perceived surveillance. This suggests that some autonomy-related risks are interpretive and may be partly buffered by technological understanding, whereas others are experiential and arise from the monitoring logic of the technology itself, especially its intrusion into private boundaries, everyday rhythms, and subjective comfort [5,32].

### Conclusions

This study shows that algorithmic care can reduce older adults’ perceived autonomy in community smart older adult care settings by increasing decisional substitution and perceived surveillance. It also shows that digital literacy is a selective rather than universal buffer: it weakens decisional substitution but does not significantly reduce perceived surveillance. These findings move smart older adult care research beyond technology acceptance by showing how algorithmic systems can reorganize older adults’ judgment, participation, and psychological boundaries in everyday care.

For smart older adult care to support rather than replace older adults, system design and service governance should preserve choice, clarify monitoring boundaries, strengthen opt-out possibilities, and keep human discussion within the care process. System prompts should rely more on suggestive than commanding logic, and care workers or family members should avoid treating algorithmic outputs as final decisions.

## Supplementary material

10.2196/94559 Multimedia Appendix 1 participant characteristics, first-order themes identified from semi-structured interviews, and measurement items.

## Abbreviations

HTMT: heterotrait-monotrait ratio
