# Supplementary material for: Perceived Algorithmic Care Intensity and Older Adults’ Autonomy in Community Smart Care: Mixed Methods Study
Source: JMIR Aging. 2026 Jul 17;9:e94559. doi: 10.2196/94559 (PMC13378906; doi:10.2196/94559)
Supplement: Multimedia Appendix 1 [file aging-v9-e94559-s001.docx]

Table S1. Participant Characteristics

| **Participant ID** | **Age** | **Gender** | **Education** | **Living Arrangement** | **Health Status** | **Smart Eldercare Services/Devices Used** | **Digital Literacy** |
| --- | --- | --- | --- | --- | --- | --- | --- |
| P01 | 62 | Female | Junior high school | Living with spouse | Hypertension | Community health platform, smart wristband | Medium |
| P02 | 68 | Male | High school | Living alone | Diabetes | Emergency call device, blood pressure monitor | Low |
| P03 | 71 | Female | Primary school | Living with children | Arthritis | Community follow-up system | Low |
| P04 | 66 | Male | Junior college | Living with spouse | None | Smart wristband, community app | High |
| P05 | 74 | Female | Junior high school | Living alone | Hypertension, heart disease | Home sensing device, emergency call device | Low |
| P06 | 69 | Male | High school | Living with spouse | Diabetes | Blood glucose monitor, community platform | Medium |
| P07 | 77 | Female | Primary school | Living alone | Hypertension | Fall detection device, emergency call device | Low |
| P08 | 64 | Male | Bachelor’s degree | Living with spouse | None | Smart watch, community health app | High |
| P09 | 72 | Female | High school | Living with spouse | Osteoporosis | Home sensing device, community platform | Medium |
| P10 | 80 | Male | Primary school | Living with children | Coronary heart disease | Remote consultation platform, emergency call device | Low |
| P11 | 67 | Female | Junior college | Living alone | None | Community app, sleep monitoring device | High |
| P12 | 75 | Male | Junior high school | Living with spouse | Hypertension | Fall detection device, home sensor | Low |
| P13 | 63 | Female | Bachelor’s degree | Living with spouse | None | Smart wristband, community health platform | High |
| P14 | 78 | Male | Junior high school | Living alone | Diabetes, hypertension | Emergency call device, community follow-up system | Low |
| P15 | 70 | Female | High school | Living with children | Hypertension | Home sensing device, community app | Medium |

Table S2. First-Order Themes Identified from Semi-Structured Interviews and Their Links to Study Constructs

| **First Order Theme** | **Core Meaning** | **Illustrative Quote** | **Related Variable / Research Use** |
| --- | --- | --- | --- |
| Safety and convenience benefits | Smart eldercare improves safety and daily convenience | “I live alone, and what I use most are the emergency call device and blood pressure monitor. Honestly, they do make me feel safer, especially at night when I do not feel well.” (P02) | Shows the practical value of smart eldercare and provides background for the study context |
| Deep system involvement in care | Older adults distinguish between information recording tools and systems that actively make judgments | “It reminds me too often… After a while, it feels like the system knows better than I do how I should arrange things.” (P01) | Corresponds to perceived algorithmic care intensity and informs vignette development |
| Weakened decision-making role | Stronger system involvement compresses personal judgment and participation | “Many things are no longer decided by me first. I look at what the machine says first.” (summarized from P02, P06, P14) | Corresponds to decisional substitution and provides experiential support for H2 and H4a |
| Being continuously seen and leaving traces | Older adults feel recorded, observed, and followed | “It feels like there are always eyes in the house. It is not a camera, but you know the system keeps track of many of your movements.” (P05) | Corresponds to perceived surveillance and informs item refinement for H3 and H4b |
| Boundary disruption and changing relationships | Information transmission weakens personal boundaries and increases outside intervention | “Some situations I could have handled myself, but once the system sends it out, my family gets involved immediately.” (P09) | Extends the meaning of perceived surveillance and reflects changes in care relationships |
| Reduced autonomy and control | Deep technological involvement weakens one’s sense of agency and personal rhythm | “If a good life is defined entirely by the system, then my own rhythm and preferences will gradually be squeezed out.” (P15) | Corresponds to perceived autonomy and informs revision of the outcome variable |

**Measurement Items**

**I. Perceived Algorithmic Care Intensity** (Jaana et al., 2025; Neves et al., 2024)

This care system actively provides reminders or suggestions based on the information it collects.

My daily care arrangements rely to a large extent on the system’s recognition and judgment.

Much of the care-related information is processed by the system before it is fed back to me.

**II. Decisional Substitution**

Many care-related decisions feel as if the system makes the judgment before I do.

Under this type of care, I often just follow what the system arranges.

I feel that my participation in care-related decisions is rather limited.

At times, I feel that the system decides what I should do more than I do.

**III. Perceived Surveillance**

I feel that my daily life is constantly being observed by the system.

This type of care makes me feel that I am always being recorded.

I worry that the system knows too much about the details of my life.

I feel that my physical condition and daily routines are continuously tracked by the system.

**IV. Perceived Autonomy**

Under this type of care, I still feel that I can take charge of important matters related to myself.

I feel that I still have sufficient choice in the care process.

This type of care does not weaken my sense of control over my own life.

Even with system involvement, I still feel that many things are ultimately decided by me.

**V. Digital Literacy**

I know how to use digital devices to obtain health related information.

I am able to understand the health or care information provided by digital platforms.

I am confident in judging whether information on digital platforms is reliable.

I know how to use digital devices to solve problems related to health management.

When the system provides suggestions or reminders, I can usually understand what they mean.

I am able to use digital tools to support my daily health management.
